# Supplementary material for: Administration of Bacteriophages via Nebulization during Mechanical Ventilation: In Vitro Study and Lung Deposition in Macaques
Source: Viruses. 2023 Feb 22;15(3):602. doi: 10.3390/v15030602 (PMC10051375; doi:10.3390/v15030602)
Supplement: Supplementary file 1 [file viruses-15-00602-s001.zip › Supplementary Data S1.pdf]

PP1450			Plaque spot assay of serial dilutions			
			Phenotype			
			PLAQUE FORMING UNITS		LYSIS w/o PFU	NO LYSIS
			EOP ≥ 0.005	0.005 > EOP > 0	EOP = 0	EOP = 0
**Broth microdilution**	MIC (PFU/mL)	MIC ≤ 1 × 10^7	16	1	1	0
		MIC < 1 × 10^9	1	3	0	0
		MIC ≥ 1 × 10^9	0	3	13	3

PP1777			Plaque spot assay of serial dilutions			
			Phenotype			
			PLAQUE FORMING UNITS		LYSIS w/o PFU	NO LYSIS
			EOP ≥ 0.005	0.005 > EOP > 0	EOP = 0	EOP = 0
**Broth microdilution**	MIC (PFU/mL)	MIC ≤ 1 × 10^7	15	3	0	0
		MIC < 1 × 10^9	1	1	2	0
		MIC ≥ 1 × 10^9	0	2	10	7

PP1792			Plaque spot assay of serial dilutions			
			Phenotype			
			PLAQUE FORMING UNITS		LYSIS w/o PFU	NO LYSIS
			EOP ≥ 0.005	0.005 > EOP > 0	EOP = 0	EOP = 0
**Broth microdilution**	MIC (PFU/mL)	MIC ≤ 1 × 10^7	13	0	0	0
		MIC < 1 × 10^9	2	0	5	0
		MIC ≥ 1 × 10^9	3	1	14	3

PP1797			Plaque spot assay of serial dilutions			
			Phenotype			
			PLAQUE FORMING UNITS		LYSIS w/o PFU	NO LYSIS
			EOP ≥ 0.005	0.005 > EOP > 0	EOP = 0	EOP = 0
**Broth microdilution**	MIC (PFU/mL)	MIC ≤ 1 × 10^7	3	1	2	0
		MIC < 1 × 10^9	1	4	9	0
		MIC ≥ 1 × 10^9	2	2	15	2

Supplementary Data S1: Phage activities on the international *P. aeruginosa* reference panel. For each phage, the number of strains, out of 41, falling into each category-are indicated. The colored cells refer to the susceptibility classification proposed in Figure 4. The strain was considered as resistant “R” when no lysis was observed on the spot plaque assay or when the MIC was above 1 × 10^9 PFU/mL. The strain was defined as susceptible “S” as long as the MIC was below 1x10^7 PFU/mL, and when the MIC was between 1 × 10^7 and 1 × 10^9 PFU/mL with an EOP above 0.005. The strain was defined as susceptible at Increased exposure “I” when MIC was between 1 × 10^7 and 1 × 10^9 PFU/mL and an EOP below 0.005 or when a lysis of the spot without PFU was observed on the plaque spot assay.
