# Supplementary material for: Administration of Bacteriophages via Nebulization during Mechanical Ventilation: In Vitro Study and Lung Deposition in Macaques
Source: Viruses. 2023 Feb 22;15(3):602. doi: 10.3390/v15030602 (PMC10051375; doi:10.3390/v15030602)
Supplement: Supplementary file 1 [file viruses-15-00602-s001.zip › Supplementary Data S2.pdf]

Supplementary Data S2: Radioactive tracing method for modeling anti-pseudomonal phage mix aerosol

S2-1. Radioactive tracing method

Extraction and quantification of phages presents technical limitations for *in vitro* aerosol collection by filtering method and *in vivo* lung deposition study. A comparative study between two aerosols was conducted to validate the use of di-ethylene-triamine-penta-acetic acid labeled with technetium 99m (^{99m}Tc-DTPA, Technescan™ DTPA, Curium Pharma, France) as a radioactive aerosol model for phages aerosol.

Static mesh nebulizer (prototype, DTFmedical, Saint Etienne, France) was used to nebulize two solutions: the solution A was the AP-Phage mix, alone in 0.9% NaCl and the solution B was the A completed with the radioactive tracer ^{99m}Tc-DTPA.

Thus, the mass-weighted particle-size distribution of the both AP-Phage mix and ^{99m}Tc-DTPA within the two aerosols were evaluated according to European Pharmacopoeia (Eur. Pharm.) method [48]. Next-generation cascade impactor (NGI, Copley Scientific, Nottingham, UK) cooled at 5°C and operated at an aspiration flow rate of 15 L/min was used to collected the two aerosols by 14.1 µm - 0.98 µm diameter cut-off scale (Figure S2a). After nebulization, each impaction stage and terminal filter were rinsed with 10 mL of 150 mM KCL solution. For aerosol A, mass distribution was calculated based on phage titers (spot assay). For aerosol B, it was calculated based on ^{99m}Tc activity determined by imaging collection cups during 120 s with a single head gamma camera (E-Cam, Siemens Healthcare, Munich, Germany). Distributions were determined according to Eur. Pharm. requirements.

S2-2. Particle size distribution of bacteriophage aerosol mix to radiotracer

Figure S1b shows the mass distribution of the AP-Phage mix aerosol (phage titers) and of the radioactivity contained in the second aerosol with phages (^{99m}Tc-DTPA activities). Distributions were globally similar, in terms of the percent deposited according to the cut-off diameters of the cascade impactor. However, a limitation for phage titration at the two-sides of the distribution do not allow statistical validation of equivalence for the 3.3µm-cut-off diameter, due to a plausible instability of phage at low concentration in the cup's samples (<1.0 × 10^5 PFU).

The mass median aerodynamic diameter (MMAD) of phages aerosol was 3.4 µm and which of the radioactive tracer aerosol (nebulized with phages) was 3.1 µm (Figure S1c and d). The respirable fractions (RF) of aerosol were similar: the RF< 5 µm (pulmonary deposition requirement) was 83 ± 1% for phages alone and 83 ± 3% for radioactive tracer; RF < 2 µm (alveolar deposition requirement) was 9 ± 5% for phages alone and 13 ± 2% for radioactive tracer.

Although a no-perfect equivalence of the two aerosols, the lung deposition pattern should be relatively the same for the two aerosols (no clinical difference between a 3.1 µm aerosol and a 3.4 µm aerosol). Results suppose that the ^{99m}Tc-DTPA can be used with AP-Phage mix as aerosol model to mimic and study the aerodynamics characteristics of AP-Phage aerosol alone.

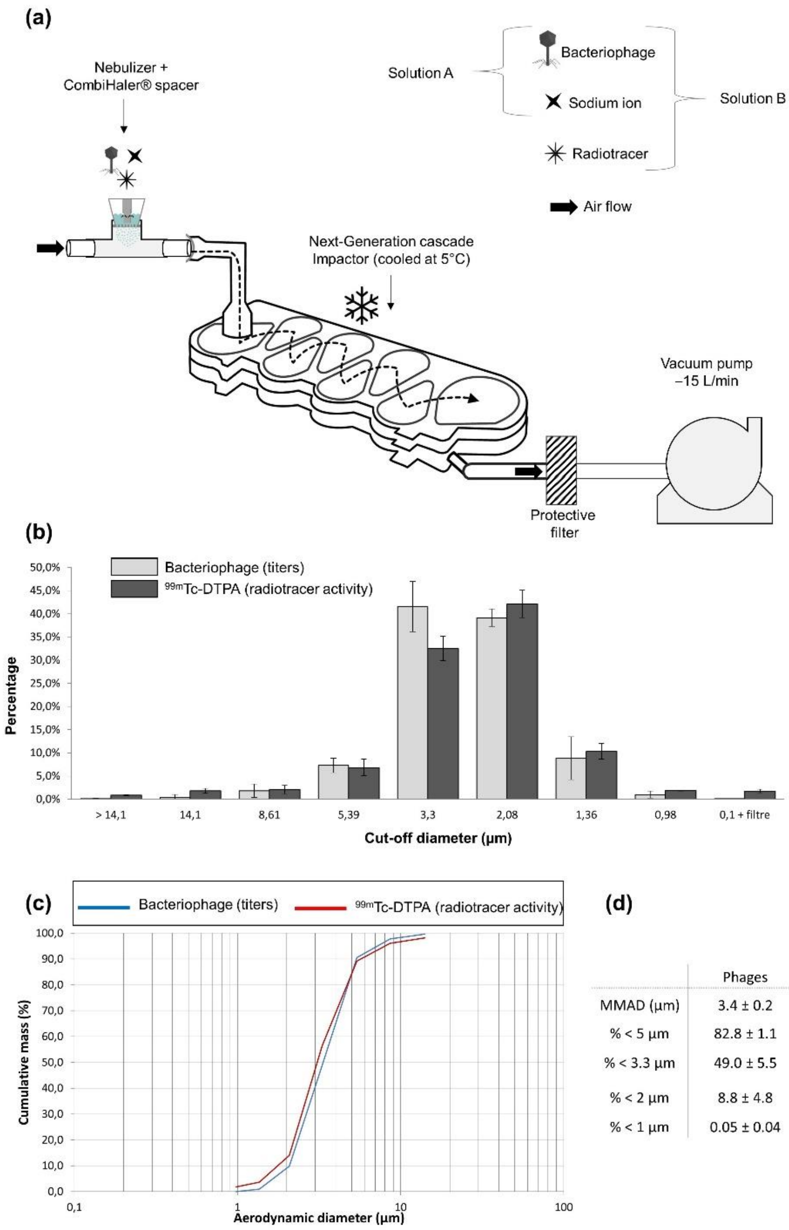

	Phages	^{99m}Tc-DTPA
MMAD (µm)	3.4 ± 0.2	3.1 ± 0.1
% < 5 µm	82.8 ± 1.1	83.1 ± 2.5
% < 3.3 µm	49.0 ± 5.5	56.5 ± 4.1
% < 2 µm	8.8 ± 4.8	12.9 ± 1.5
% < 1 µm	0.05 ± 0.04	1.9 ± 0.4

Figure Supplementary Data S2: Radioactive tracing method for modeling anti-pseudomonal phage mix aerosol. (a) The method used a next-generation cascade impactor (NGI) operating at −15L/min (aspiration airflow rate) to sample and collect aerosols based on the cut-off diameter of each stage. Two solutions were nebulized with the static mesh prototype nebulizer (connected to a T-piece). Solution A contained phage mix alone in 0.9% NaCl, and solution B comprised phage mix in 0.9% NaCl with the radioactive tracer ^{99m}Tc-DTPA. On each stage, phages were titrated by a spot assay method and radiotracer activities were quantified by a gamma camera. (b) The mass distribution of the AP-phage mix or ^{99m}Tc-DTPA was expressed in % of total mass deposited in the cascade impactor NGI according to European Pharmacopoeia guideline [48]. (c) The mass-cumulative distribution is expressed as the percent of phages mass or ^{99m}Tc-DTPA titers or quantities. (d) Aerodynamics parameters extracted from mass cumulative distribution: MMAD for mass median aerodynamic diameter (µm).
